# Apoptosis Inhibitor 5: A Multifaceted Regulator of Cell Fate

**DOI:** 10.3390/biom14010136

**Published:** 2024-01-22

**Authors:** Hafsia Abbas, Dalia Kheira Derkaoui, Louise Jeammet, Emilie Adicéam, Jérôme Tiollier, Hélène Sicard, Thorsten Braun, Jean-Luc Poyet

**Affiliations:** 1 Université Oran 1, Ahmed Ben Bella, Oran 31000, Algeria; latifaabbas472@yahoo.com (H.A.); derkaouidalia@yahoo.fr (D.K.D.); 2 Jalon Therapeutics, 75010 Paris, France; louise.jeammet@jalontx.com (L.J.); jerome.tiollier@jalontx.com (J.T.); helene.sicard@jalontx.com (H.S.); 3 Laboratoire de Transfert des Leucémies, EA3518, Institut de Recherche Saint Louis, Hôpital Saint Louis, Université de Paris, 75010 Paris, France; thorsten.braun@aphp.fr; 4 AP-HP, Service d’Hématologie Clinique, Hôpital Avicenne, Université Paris XIII, 93000 Bobigny, France; 5 OPALE Carnot Institute, The Organization for Partnerships in Leukemia, Hôpital Saint-Louis, 75010 Paris, France; 6 INSERM UMRS976, Institut de Recherche Saint Louis, Hôpital Saint Louis, 75010 Paris, France; 7 Université Paris Cité, 75015 Paris, France

**Keywords:** apoptosis inhibitor 5, apoptosis, cancer, therapeutic target

## Abstract

Apoptosis, or programmed cell death, is a fundamental process that maintains tissue homeostasis, eliminates damaged or infected cells, and plays a crucial role in various biological phenomena. The deregulation of apoptosis is involved in many human diseases, including cancer. One of the emerging players in the intricate regulatory network of apoptosis is apoptosis inhibitor 5 (API5), also called AAC-11 (anti-apoptosis clone 11) or FIF (fibroblast growth factor-2 interacting factor). While it may not have yet the same level of notoriety as some other cancer-associated proteins, API5 has garnered increasing attention in the cancer field in recent years, as elevated API5 levels are often associated with aggressive tumor behavior, resistance to therapy, and poor patient prognosis. This review aims to shed light on the multifaceted functions and regulatory mechanisms of API5 in cell fate decisions as well as its interest as therapeutic target in cancer.

## 1. Introduction

Apoptosis is a highly regulated form of programmed cell death that plays a crucial role in various physiological and pathological conditions, including development, tissue homeostasis, and the removal of damaged or unnecessary cells [1,2,3,4]. It is now well established that defects in the apoptotic pathways are closely related to both oncogenesis and cancer treatments resistance [5,6,7,8]. Understanding the molecular mechanisms regulating apoptosis is therefore of crucial importance for the identification of specific targets for anticancer therapies. Apoptosis execution relies on the highly regulated activation of a group of cysteine proteases called caspases that specifically cleave a series of substrates, resulting in cell death [9,10,11]. Caspases are synthesized as inert zymogens that are activated through two distinct, but interconnected, pathways, called the intrinsic or extrinsic pathways, in which apoptotic stimuli trigger the activation of the so-called initiator caspases (such as caspase-2, -8, -9, and -10) which, in turn, proteolytically cleave and activate effector (or executioner) caspases (caspase-3, -6, and -7) [12,13]. When activated, the effector caspases specifically cleave a broad spectrum of cellular targets, ultimately leading to cell death.

The extrinsic pathway is initiated by the activation of death receptors, upon binding of their cognate ligands and subsequent recruitment at the level of the cytoplasmic region of the death receptors of death domain-containing adaptor proteins [2]. This results in the formation of a death-inducing signaling complex (DISC), which can in turn recruit and activate caspase-8 via oligomerization. Death receptor-mediated apoptosis can be inhibited by a proteolytically inactive homolog of caspase-8, called cellular FLICE inhibitory protein (cFLIP), which can be recruited to the DISC, forming a proteolytically inactive heterodimer with caspase-8 [14].

The intrinsic pathway, also known as the mitochondrial pathway, proceeds through the induction of the mitochondrial outer membrane permeabilization (MOMP) and the subsequent release in the cytoplasm of numerous proapoptotic mitochondrial constituents [15]. Among these, cytochrome *c* promotes the oligomerization of apoptotic protease-activating factor-1 (APAF-1), triggering the formation of the apoptosome and dimerization-induced activation of caspase-9 [16]. The intrinsic pathway is intricately regulated by pro- and anti-apoptotic B-cell lymphoma-2 (Bcl-2) family members, which consist of evolutionarily conserved proteins that share at least one Bcl-2 homology (BH) domain [17].

Although the connection between the number of genetic mutations and cancer is complex, the tumorigenesis process relies on both the activation of oncogenes that stimulate cancer cells proliferation and survival, as well as the inactivation of tumor suppressor genes that hold cellular proliferation in check [18]. To date, a wide variety of oncogenes and tumor suppressor genes involved in the regulation of pro- or anti-apoptotic signals have been discovered. Variations in the expression of these genes, or their mutation, can contribute to tumor initiation, progression or resistance to treatment. Consequently, a number of therapeutic approaches have been developed to overcome cell death resistance through the pharmacological manipulation of various apoptosis signaling networks. Current main therapeutic strategies include either inhibiting antiapoptotic regulators or stimulating proapoptotic factors [19,20]. For instance, a number of inhibitors of antiapoptotic Bcl-2 family members, which are known to be overexpressed in numerous cancers, are now used in clinics. These include the Bcl2-selective BH3-mimetic Venetoclax [21], which is currently used for the treatment of chronic lymphocytic leukemia, small lymphocytic lymphoma, or acute myeloid leukemia, or the myeloid cell leukemia-1 (Mcl-1) inhibitors S63845, AMG-176, and AZD5991 [22,23,24].

Among the cell death regulators is API5 (apoptosis inhibitor-5), also known as AAC-11 (anti-apoptosis clone 11 or FIF (fibroblast growth factor-2 interacting factor)), a 55 kDa nuclear scaffold protein initially discovered as a negative regulator of apoptosis upon nutritional stress conditions [25]. API5 has emerged as a key player in the context of cancer as its overexpression has been associated with aggressive tumor behavior, resistance to treatment, and poor prognosis [26,27,28,29,30,31,32,33,34,35]. Furthermore, recent observations indicate that API5 influence extends far beyond apoptosis regulation, making this intriguing protein a versatile regulator of cell fate with diverse functions ranging from anti-apoptosis to metastasis, cell cycle control, mRNA export, and TLR4-dependent activation and maturation of antigen presenting cells. API5’s intricate involvement in these critical cellular processes underscores its significance in both health and disease, particularly in cancer biology. This review aims to explore the biological functions of API5, shedding light on its molecular mechanisms, interactions, and potential therapeutic applications.

## 2. Structure and Binding Partners of API5

**Figure 1:**
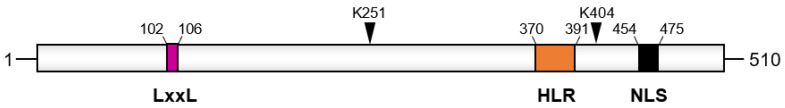
Domain organization of API5. The LxxLL motif, the acetylation site (K251), the SUMOylation site (K404), the heptad leucine repeat (HLR), and the nuclear localization domain (NLS) are shown. Numbers indicate amino acids positions.

API5 is an evolutionarily conserved, mostly nuclear protein found in various organisms, from protists to animals and plants [36]. It belongs to the API5 family, whose members contain the so-called API5 domain (Pfam ID PF05918), a domain notably absent in proteins encoded by eubacteria, archaebacteria, and viruses’ genomes, but which is encoded by genomes from several ancient eukaryotic taxonomic groups [37]. Alignment analyses of API5 orthologs across species, vertebrates, and invertebrates, indicate a high level of conservation in the sequence and protein domains [38,39,40]. It is well established that protein domains are functional and structural units within proteins that can be added, rearranged, or combined through evolution to create novel functionalities and facilitate the creation of complex biological systems [41,42,43]. In that sense, API5 is characterized by a unique domain structure, which comprises multiple functional and protein–protein interaction modules. These conserved modules include an LxxLL motif, a heptad leucine repeat region, and a nuclear localization sequence (Figure 1).

**Figure 2:**
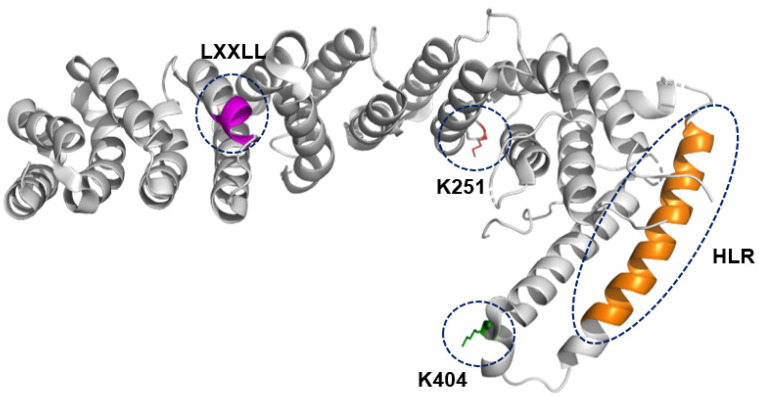
Structural representation of API5. The LxxLL motif (in magenta), the acetylation site (K251; in red), the SUMOylation site (K404; in green), and the heptad leucine repeat (HLR; in orange) are shown.

Structural analysis indicates that API5 presents an elongated, all-helical structure (Figure 2), with an N-terminal HEAT repeat segment and C-terminal ARM (Armadillo)-like repeat regions, which have also been demonstrated to act as protein–protein interaction modules [40]. Elongated structures in repeat proteins are known to favor interactions with multiple binding partners [44,45,46]. It is therefore likely that API5 can function as a scaffold protein, providing a platform for signaling molecules to assemble into functional units and regulating the spatial–temporal organization of various signaling pathways.

As of today, a number of API5 interacting proteins that are implicated in various cellular functions have been described (Table 1 and paragraphs below). The first described API5 interactor is the high-molecular-weight forms of basic fibroblast growth factor (HMW FGF-2), which binds to API5 via two specific regions, located at the N-terminal (residues 96–107) and a C-terminal (residues 274–311) parts of the protein. Several other API5 partners have since been identified, which are involved in an array of cellular functions such as apoptosis, cell survival, immune response, transcription, RNA export, or chromatin remodeling (Table 1). It is interesting to note that most of API5 interactors are known to be components of high-molecular-weight multiprotein complexes, again suggesting that API5, which has no known enzymatic functions, might provide a molecular hub for the docking of signaling molecules.

## 3. Physiological Functions of API5

### 3.1. Anti-Apoptotic Functions of API5

API5 has been originally identified as a survival factor whose expression prevents apoptosis in the absence of serum [25]. In line with these results, a study carried out in a cervical cancer cell line showed that overexpression of AAC-11 conferred a survival advantage to the cells in serum-free medium [29]. Since then, various studies have highlighted a critical role for API5 in several apoptotic pathways, establishing API5 as a multifunctional cell death regulator.

#### 3.1.1. Inhibition of E2F1 (E2F Transcription Factor 1)-Induced Apoptosis

**Figure 3:**
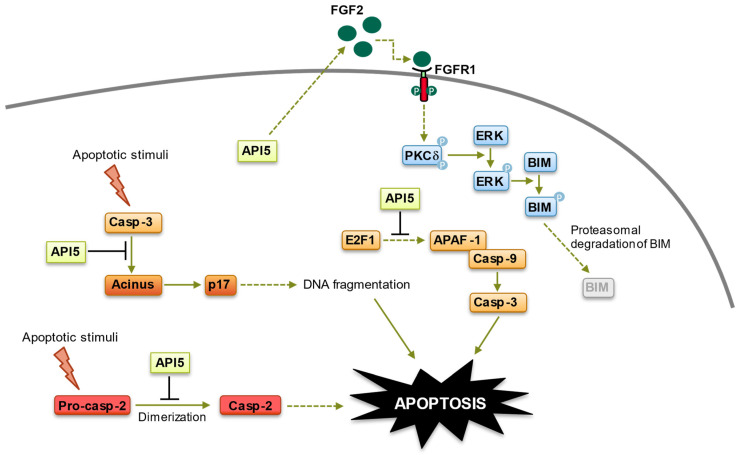
Anti-apoptotic functions of API5. API5 regulation of apoptosis takes place at four levels: (1) API5 inhibits E2F1-induced apoptosis. (2) API5 inhibits Acinus-induced apoptotic DNA fragmentation. (3) API5 inhibits caspase-2 activation. (4) API5 upregulates FGF2/FGFR1 signaling, leading to BIM degradation. Of note, API5-mediated activation of FGFR1 signaling, which triggers proteasome-dependent degradation of BIM, is also involved in the chemo- and immune-resistance of cancer cells.

E2F1 is a crucial target of the retinoblastoma tumor suppressor protein (pRb) [56]. Its most well-documented function is the transcriptional regulation of numerous genes pivotal for cell cycle progression [57]. The activation of E2F1 is prompted by mitogenic signals, initiating the transcription of genes responsible for G1/S phase transition and DNA synthesis, such as *cyclin E*, *cyclin A*, *Cdk2*, *cdc25*, and *SKP2* [58,59]. As the pRb pathway is inactive in many tumor cells, this can lead to the dysregulation of E2F1 activity, resulting in uncontrolled cell proliferation. On the other hand, E2F1 can also induce the stabilization and activation of the tumor suppressor p53 and directly activate the transcription of pro-apoptotic genes, such as *Fas* (*CD95/APO-1*), *APAF-1*, and *caspase-3* in response to DNA damage stimuli, subsequently leading to apoptosis [60,61,62,63]. Consequently, due to the dual role of E2F-1 activation, the Rb/E2F1 apoptotic pathways are disrupted in many human tumors through the loss of p53 as well as mechanisms that are yet to be elucidated [64]. This disruption upsets the usual balance between apoptosis and proliferation, allowing unchecked proliferation without the protective mechanisms of apoptosis to hinder tumorigenesis. Consequently, identifying factors that inhibit E2F-1-induced apoptosis is of great interest as they might constitute excellent targets for therapeutic intervention. Using a *Drosophila*-based genetic approach, Morris and colleagues demonstrated that API5 plays a pivotal role in dE2F1-induced apoptosis [39]. While the precise mechanisms involved are still unknown, a careful analysis of API5 regulation of E2F1-mediated apoptosis, which is conserved from flies to humans, revealed that API5 functions downstream of E2F1, suppressing E2F1-dependent apoptosis without impeding E2F-dependent transcription [39] (Figure 3). The strong genetic interaction between E2F1 and API5 suggests that elevated levels of API5 may be selected during tumorigenesis to allow cells with deregulated E2F1 activity to survive under suboptimal conditions by limiting the extent of E2F1-dependent cell death. Interestingly, a peptide preventing API5 interaction with its partners has been demonstrated to potentiate E2F1-induced apoptosis in tumor cells [65], and supporting the inhibition of API5 function as a new strategy to induce E2F1-dependant cell death might offer a possible mechanism for antitumor exploitation (see below).

#### 3.1.2. Inhibition of Acinus (Apoptotic Chromatin Condensation Inducer in the Nucleus)-Induced Apoptotic DNA Fragmentation

While the nuclear factor Acinus has been originally described as essential for apoptotic chromatin condensation [66], subsequent observations have suggested a role for Acinus in apoptotic DNA fragmentation [65,67]. Mechanistically, Acinus, which is expressed in different isoforms, is activated upon proteolytic cleavage by caspase-3, resulting in the formation of a truncated, active form called p17 that induces chromatin condensation in an in vitro system by utilizing permeabilized cells [66]. The caspase-mediated cleavage of Acinus has also been involved in erythroid maturation, which requires the transient, non-cell lethal activation of several caspases [68]. Finally, subsequent experiments have revealed a role for Acinus in pre-mRNA processing. Indeed, Acinus is a crucial component of the apoptosis and splicing-associated protein complex (ASAP), which regulates the activity of the exon junction complex (EJC), an RNA-binding multi-protein platform with critical functions in post-transcriptional gene regulation [69,70,71,72]. Therefore, Acinus might be involved in both apoptosis and pre-mRNA processing. Using a yeast two-hybrid strategy, we have demonstrated that Acinus is a binding partner of API5. Interestingly, API5 binding to Acinus prevents Acinus-mediated apoptotic DNA fragmentation by protecting Acinus from caspase-3 cleavage and the subsequent generation of the p17 active fragment [65] (Figure 3). However, how exactly API5 protects Acinus apoptotic cleavage is still unknown, and it might be worth noting that API5 binds to a central region of Acinus (residues 840–918) that is relatively close to the caspase-3 cleavage sites (987 and 1093). It is therefore possible that, upon binding, API5 might shield caspase-3 cleavage sites of Acinus. In any case, these observations indicate a central role for API5 in apoptotic DNA fragmentation.

#### 3.1.3. Inhibition of Caspase-2 Activation

While caspase-2 is the most evolutionarily conserved member of the mammalian caspase family, its physiological functions have been a matter of considerable debate. The enzyme contains a prodomain containing the caspase-recruitment domain (CARD) followed by two catalytic active domains: p19 and p12. The CARD engages, upon interaction with cognate adaptor proteins, in the dimerization-induced activation of caspase-2 [73]. Caspase-2 can be activated in response to various stimuli, such as heat shock or DNA damage, and initiate apoptosis through the cleavage of a number of cellular substrates such as Bid, resulting in mitochondrial outer membrane permeabilization (MOMP) and leading to cell demolition [74]. Furthermore, numerous observations have established that, beside apoptosis, caspase-2 is also involved in a range of diverse cellular functions. These functions include cell cycle regulation, DNA repair, lipid sensing, tumor suppressor, metabolic regulation, the regulation of oxidant levels in cells, and aging (see [74] for review). Furthermore, in the liver, caspase-2 plays an essential role in the pathogenesis of non-alcoholic steatohepatitis [75]. In the nervous system, caspase-2 is involved in synaptic plasticity and cognitive flexibility, and in several neuropathological mechanisms, such as neonatal brain lesions, retinal ischemia, and the synaptotoxic effects of β-amyloid peptide and tauopathies (see [76] for review). Taking advantage that caspase-2 acts as an initiator caspase for pore-forming toxin-mediated apoptosis in various cells [77], Imre and colleagues performed a mass spectrometric analysis of active caspase-2-containing complexes induced by *Staphylococcus aureus* α-toxin to identify proteins that regulate caspase-2 activation in this setting [51]. Using HeLa cells, Imre et al. demonstrated that API5 directly interacts with caspase-2 and negatively modulates its activation [51]. Mechanistically, API5 directly binds to the CARD domain of caspase-2, but not to the CARD domain of other caspases, and this binding prevents the CARD-mediated dimerization/activation of caspase-2 (Figure 3). In line with these observations, the shRNA-mediated downregulation of API5 enhanced caspase-2 dimerization/activation, sensitizing cells to caspase-2-mediated apoptosis and resulting in significant apoptosis sensitization of HeLa cells to α-toxin [51]. Of note, API5 could not inhibit processed caspase-2 activity [51]. At this moment, the impact of API5 post-translational modifications (see below) on its ability to interact with caspase-2 or the subcellular localization and the precise stoichiometry of the API5–caspase-2 complex remain to be determined. Nevertheless, these interesting results establish API5 as a novel endogenous inhibitor of caspase-2 and indicate that API5 modulation of apoptosis stems, at least in part, from its ability to inhibit caspase-2 activation.

#### 3.1.4. Fibroblast Growth Factor Receptor 1 (FGFR1)/Extracellular Signal-Regulated Kinase 1/2 (ERK1/2) Signaling-Mediated Degradation of BIM

The Fibroblast Growth Factor (FGF) family comprises secreted signaling proteins (secreted FGFs) that act via interaction with four signaling tyrosine kinase FGF receptors (FGFRs). Among the secreted FGFs, fibroblast growth factor-2 (FGF-2), upon binding to FGFR 1-4 and activation of various signaling pathways such as RAS–mitogen-activated protein kinase (MAPK) and phosphatidylinositol-4,5-bisphosphate 3-kinase (PI3K)-AKT signaling, plays a crucial role in cell development, differentiation, regeneration, senescence, proliferation, and migration, as well as in tumor progression and malignancy [78,79].

Several studies have revealed that API5 is a direct interactor of FGF-2 and that API5 and FGF-2 are concomitantly upregulated in a number of malignancies [30,47,48]. Using various cellular settings, Tae Woo Kim’s group demonstrated that API5 actually upregulates FGF-2 expression [31,80]. Indeed, a clear correlation between API5 and FGF-2 expression was observed in an array of cancer cells, and forced expression or siRNA-mediated inhibition of API5 resulted in concomitant FGF-2 expression increase or decrease, respectively [31,33,80]. In turn, API5-mediated upregulation of FGF-2 expression triggered the FGFR1/PKCδ/ERK pathway, leading to the degradation of the proapoptotic B-cell lymphoma 2 (Bcl-2) protein family member BIM [31,33,80,81] (Figure 3). This degradation has two crucial consequences: (1) it allows an in vivo immune escape of cancer cells via resistance to antigen-specific T-cell-induced apoptosis (see below) [31] and (2) it mediates chemoresistance of cancer cells to cisplatin [80] (Figure 3). Importantly, the API5/FGFR1/ERK/BIM axis appears to be conserved in multiple cancers [80]. These observations are of great interest as, because of its multiple roles in the acquisition of a complex malignant phenotype, the ERK pathway represents an attractive target for the development of anticancer drugs [82]. Furthermore, it is now well accepted that BIM is an important regulator of tumorigenesis through activities as a tumor suppressor, tumor metastasis, and tumor cell survival and has become the focus of intense interest as a potential target for cancer chemotherapy [83]. Therefore, controlling ERK activity as well as BIM cellular levels by means of API5 targeting might open new therapeutic options for the treatment of aggressive, chemo-refractory cancers.

### 3.2. Cell Cycle Regulation Functions of API5

Besides preventing E2F1-mediated apoptosis, API5 has also been shown to modulate E2F1 control of the G1/S transition phase [84]. E2F1 plays an important role in G1/S cell cycle phase transition, as it modulates the expression of several genes related to DNA synthesis and cell cycle, resulting in cell proliferation. While in non-proliferating cells, the hypo-phosphorylated form of the pocket protein pRb prevents E2F1 transcriptional activity, the dissociation of E2F1 from pRb protein permits the transactivation of genes such as *cyclin A*, *cyclin E*, *c-myb*, *cdc2*, *PCNA*, and *thymidine kinase*, thus committing cells to S-phase progression [85]. In their work Garcia-Rove Navarro and colleagues demonstrated that, like E2F1, the expression levels of API5 are regulated periodically during the cell cycle, with a higher expression during the G1 phase, a stabilization during the G1/S transition, and a decrease from the G2 to the G2/M phase [84]. RNA interference analyses showed that API5 depletion reduced the expression of E2F1 target genes, such as *cyclin A*, *cyclin E*, *cyclin D1*, or *Cdk2*, leading to G1 cell cycle arrest and cell cycle delay. Mechanistically, API5 positively regulates E2F1 transcriptional activity by increasing E2F1’s binding to its target promoters [84]. However, at this moment, the precise mode of action of API5 on E2F1 activity remains to be determined, as no direct interaction between the two proteins has been demonstrated. Interestingly, a recent study demonstrated that, like E2F1, API5 stability was positively regulated by the histone acetyltransferase p300-mediated acetylation at lysine 251 [53]. Therefore, one can envision that API5 at peak expression, during the G1- and S-phases of the cell cycle, could act as a transcription factor that modulates the transcription of cell cycle regulators. In line with this hypothesis, API5 was found to be associated with the chromatin in the nucleus [84] and previous observations indicated that API5 possesses transactivation capacities [48]. Nevertheless, no API5 target genes have been identified so far, and further work will be needed to decipher the precise role of API5 in E2F1-dependent transcription control of both G1/S- and G2/M-regulated genes. Of note, API5 has also been shown to cooperate with Estrogen Receptor α (Erα) to regulate gene expression, again arguing for a transcription-related function for API5 [26]. Recently, the impact of API5 expression in tumor cell proliferation has been clinically demonstrated in the context of cervical cancer and breast cancer [28,86]. Moreover, a role for API5 in estrogen (E2)-induced proliferation has been identified in ERα positive breast cancer cell lines [26]. Therefore, the cell cycle-related role of API5 could be particularly significant in cancer, where API5 is often overexpressed (see below) and where uncontrolled cell division is a hallmark.

### 3.3. mRNA Export Functions of API5

Transport of messenger RNA (mRNA) from the nucleus to the cytoplasm is a critical process for eukaryotic gene expression. To be exported, mRNAs associate with a wide array of co-factors and translocate mainly through the nuclear pore complex (NPC) as large ribonucleotide complexes (mRNPs) [87,88]. Numerous studies over the past decades have provided a long list of molecular components that are required for the mRNA export process. To date, the best studied mRNA export receptors’ pathways are the nuclear RNA export factor 1 (NXF1)/nuclear transport factor 2-like export 1 (NXT1) and chromosome maintenance protein 1 (CRM1), with the majority of mRNAs using the NXF1/NXT1 heterodimer route [89]. Importantly, both pathways are dysregulated in cancer and mRNA export dysregulation has been associated with various other human diseases, including neurodegeneration and viral infection, as well as aging [90,91].

Using a functional proteomics approach, the group led by Byung II Lee discovered that API5 possessed a critical role in mRNA export, through its direct interaction with various players of the mRNA export machineries [47]. Interestingly, while some of the identified proteins interacted with API5 only, several of them interacted also with FGF-2 as well as with the API5–FGF-2 complex. Among those, UAP56, an ATP-dependent RNA helicase essential for pre-mRNA splicing and mRNA export [92], was found to interact with both API5 and FGF-2, with a cooperative interaction between API5, FGF-2, and UAP56, indicating that the API5–FGF-2 complex as a whole is involved in mRNA export. As UAP56 is part of the transcription/export (TREX) complex, an essential complex for the NXF1/NXT1 pathway, as well as the CRM1 pathway [87,88], these findings suggest that the API5–FGF-2 complex is a regulatory component of both of these mRNA export pathways. Indeed, the shRNA-mediated downregulation of either API5 or FGF-2 prevented bulk mRNA export in HeLa cells, which is controlled by the TREX complex, as well as the eukaryotic translation initiation factor eIF4E-dependent mRNA export pathway, which is CRM1 dependent [47]. Furthermore, the use of a lentivirus expressing an API5-derived peptide targeting the API5–FGF-2 interaction prevented both bulk mRNA and eIF4E-dependent mRNA export pathways, indicating that API5 regulation of mRNA export machineries depend on its interaction with nuclear FGF-2 [47]. Finally, it might be worth noting that API5’s role in mRNA export seems to be evolutionary conserved, as a previous report revealed the interaction between API5 and UAP56 homologs in rice (*Oryza sativa*) [36]. Therefore, the role for API5 in mRNA export might constitute an ancestral function common to all *API5* genes.

### 3.4. Modulation of TLR4 Signaling in Dendritic Cells and Adjuvant Effect of API5

TLR4 is a member of the Toll-like receptor (TLR) family, a class of pattern recognition receptors (PRRs) which play a crucial role in pathogen recognition and activation of innate immunity [93]. TLR4 is found in the plasma membrane of neutrophils, macrophages, dendritic and endothelial cells, as well as adaptive immune cells, including T and B cells [94,95]. It is activated by structural motifs named pathogen-associated molecular patterns (PAMPs) such as lipopolysaccharide (LPS) and endogenous damage-associated molecular patterns (DAMPs), which are released upon cellular stress or tissue injury or derived from dying and necrotic cells, and which are also actively secreted from cells upon external stimulation (e.g., high-mobility group box 1 (HMGB1) protein) [96,97,98]. TLR4 activation drives the induction of inflammatory cytokines involved in innate immune responses, consequently protecting against infectious challenges and boosting adaptive immunity [99]. Interestingly, Kim et al., based on the observation that API5 expression was increased in tumor cells upon chemotherapy-induced stress, established that API5 could function as a DAMP and could directly interact with TLR4, triggering the TLR4-NF-kb pathway [52]. API5 interaction with TLR4 was specific among the TLR family and demonstrated a good affinity between the two proteins, with a Kd of approximately 430 nM. Importantly, recombinant API5 was able to induce TLR4-dependent maturation, activation, and migration of dendritic cells (DCs) upon stimulation in vitro [52]. Indeed, incubations of DCs with recombinant API5 resulted in the expression of pro-inflammatory cytokines, increased expression of maturation surface markers such as MHC class I, CD40, CD80, or CD86, as well as increased expression of migration factor CCR7, concomitantly with the activation of TLR pathway-related proteins such as ERK, AKT, or p38 and the activation of the NF-κB pathway. These DC stimulating effects were largely abolished in *TLR4*^−/−^ DCs, as compared with their WT or *TLR2*^−/−^ counterparts, confirming that API5-induced activation and maturation of DCs indeed depended on the TLR4 signaling. Interestingly, vaccination with API5-activated DCs pulsed with antigenic peptides induced antigen-specific T-cell immunity in mice associated with strong antitumor efficacy in both cancer prevention and therapeutic settings [52]. As neoantigens from cancer patients are highly personalized, the API5-stimulated DCs could therefore offer a versatile platform to prepare tailored and effective vaccine for anticancer cellular therapy. While further evaluation will be needed, Kim et al.’s results [52] therefore suggest that API5 could constitute a novel human-based adjuvant option with strong immunostimulatory properties for vaccine applications.

### 3.5. Paneth Cell Protective Action of API5 as a Putative Therapeutic Target for Crohn’s Disease

Inflammatory bowel diseases such as Crohn’s disease (CD) are characterized by chronic transmural inflammation of the gut. While CD is a heterogeneous disorder with multifactorial etiology, in which genetics and environment interact, genome-wide association studies have linked polymorphisms in the autophagy gene *ATG16L1* to Crohn’s disease susceptibility [100,101,102]. Compromised intestinal epithelial barrier functions in *ATG16L1* mutant (ATG16L1^T300A^) mice and patients with CD homozygous for the ATG16L1T^300A^ mutation have been associated with a reduced viability of Paneth cells, a subtype of intestinal epithelial cells which are important producers of antimicrobial peptides [103]. In these cells, autophagy pathways limit endoplasmic reticulum stress, preventing dysfunction and loss of viability of Paneth cells [104,105]. Unexpectedly, a recent study by Matsuzawa-Ishimoto et al. using ex vivo intestinal organoids and an *Atg16L1*-mutant mouse model identified API5 as a lymphocyte-derived protective factor of Paneth cells [106]. Indeed, liquid chromatography–mass spectrometry-based secretome analysis revealed that API5 secreted by a subset of intraepithelial lymphocytes expressing the γ and δ T-cell receptor subunits (γδ IEL) improved the viability of *Atg16L1*-deficient Paneth cells [106]. Correlative to this observation, histological analyses of terminal ileum of patients with Crohn’s disease with Atg16l1 deficiency indicated a reduction in API5+ γδ IEL cells. Importantly, the exposure of *ATG16L1^T300A^* homozygous organoids with recombinant API5 as well as the systemic injection of recombinant API5 in *Atg16L1*-deficient mice improved the viability of Paneth cells of organoids and improved disease score and overall survival in *Atg16L1*-deficient mice. While the protective mechanisms triggered by γδ IEL-secreted API5 remains to be determined, and the safety of the long-term injection of API5 in mice and humans must be carefully considered, this study demonstrates that API5 constitutes a prime therapeutic target for Crohn’s disease in the context of ATG16L1 deficiency.

### 3.6. Enhancement of Viral Replication by API5

Viruses are known to divert the functions of various host cellular proteins to facilitate their own replication. Interestingly, recent observations suggest that API5 might be involved in regulating the replication of some viruses. Indeed, the group of Lal SK has demonstrated that influenza A virus (IAV), which is known to positively modulate the intrinsic apoptotic pathway in late stages of viral infection to facilitate its propagation [107,108,109], counteracts API5 antiapoptotic properties as a means to increase its replication and propagation [50]. An investigation of the underlaying molecular mechanism revealed that IAV nucleoprotein (NP) both directly interacts with and transcriptionally downregulates API5 expression, favoring E2F1 recruitment to the *APAF-1* promoter and subsequent increase in APAF-1 expression, apoptosome formation, and caspases activation [50]. This strategy of API5 inhibition, stimulating E2F1-mediated apoptosis, thus appears to be critical to facilitate shedding and hence the dissemination of IAV. Additionally, Lal SK and colleagues’ work hints at the role of API5 in cell cycle control during IAV infection, opening avenues for further research.

Recently, in an elegant study, Deng et al. reported that API5 also acted as a negative regulator of *Avibirnavirus* infectious bursal disease virus (IBDV) replication [55]. Indeed, starting from the observation that API5 was SUMOylated at Lys404, Deng et al. showed that IBDV infection reduced SUMO2/3-conjugation of API5, and that the resulting decrease in API5-SUMOylated levels significantly favored IBDV replication [55]. API5 deSUMOylation, which triggers API5 translocation from the nucleus to the cytoplasm, was driven by the direct interaction of *Avibirnavirus* VP3 protein with API5. VP3 permits TNF receptor-associated factor 3 (TRAF3) to promote the proteasome-dependent degradation of the SUMO-conjugating enzyme UBC9, leading to API5 deSUMOylation [55]. Interestingly, the deSUMOylation of API5 prevented MDA5 (cytoplasmic RIG-I-like receptor melanoma differentiation gene 5)-dependent IFN-β production upon IBDV infection. This reduction in IFN-β production, which is known to inhibit viral replication [110], resulted in increased *Avibirnavirus* proliferation [55].

The discovery of these API5 “subversion”-based viral strategies is of interest as they offer new avenues for a better understanding of viral pathogenesis and suggest potential targets for antiviral interventions.

## 4. API5 and Cancer

### 4.1. API5 Expression and Prognosis Value

An expression analysis of API5 revealed an ubiquitous but varying expression of API5 in cancers. Of note, the human *API5* gene is located in chromosomal segment 11p12-13, in a region that is amplified in a number of cancers [111,112,113,114]. API5 has been shown to be upregulated in various cancers, such as breast cancer, colorectal cancer, cervical cancer, NSCLC, or B-cell chronic lymphoid leukemia [26,28,29,30,31,32,33,35,86,115,116]. This expression appears to be clinically relevant as it is associated with poor overall and disease-free survival as well as resistance to treatment, suggesting a potential role for API5 as a prognosis and survival marker [26,28,29,30,31,32,33,35,86]. In line with this hypothesis, Cho and colleagues have shown that API5 expression levels gradually increased during the normal-to-tumor transition of cervical carcinoma [28]. As API5 overexpression has been linked to cancer cell proliferation (see above) and survival (see below), one might envision that API5 could contribute to the development and progression of cancer.

### 4.2. API5’s Role on Cancer Metastasis, Immune Response, and Survival

**Figure 4:**
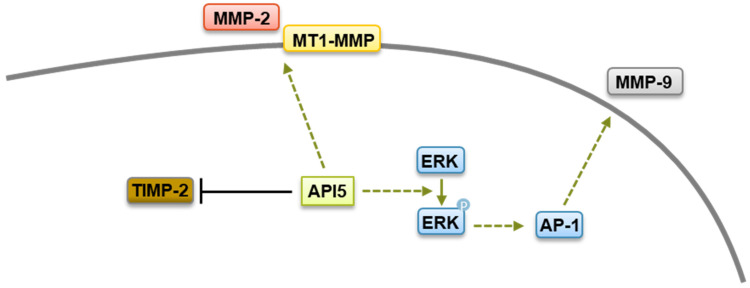
Metastasis regulation by API5. API5 increases tumor cell metastasis via upregulation of MMP-2, MMP-9, and MT1-MMP expression and downregulation of TIMP-2 levels.

The widespread and high expression of API5 in tumors suggests that API5 contributes to human malignancy. Interestingly, a recent study has shown that overexpression of API5 in breast epithelial cells induces a partial epithelial–mesenchymal transition (EMT)-like phenotype [86]. EMT is a differentiation process through which transformed epithelial cells gain the ability to invade and disseminate [117]. In line with this hypothesis, API5 expression has been demonstrated to contribute to tumor invasion and metastases in various cancer settings [26,29,34,86]. One important step in invasion is the remodeling and disassembly of the extracellular matrix and its constituents through enzymes such as matrix metalloproteinases (MMPs) [118]. MMPs are structurally related, zinc-dependent endopeptidases that have been linked to a wide variety of pathological states, including carcinogenesis, and elevated levels of MMPs correlate with unfavorable prognosis in multiple cancers [119]. An API5-forced expression increases levels of MMP-2 as well as membrane type 1 matrix metalloproteinase (MT1-MMP), with concomitant downregulation of the tissue inhibitor of MMP (TIMP-2) [29]. While the mechanism by which API5 regulates MMP-2 and MT1-MMP expression are not clear yet, API5 expression has been linked to the upregulation of the transcriptional coactivator β-catenin, which is well known to possess a crucial role in cell invasion and to regulate MMPs expression [120]. Furthermore, using other tumor settings, Song and colleagues demonstrated that API5 enhanced MMP-9 expression through an ERK-dependent regulation of activator protein 1 (AP-1) [34]. Therefore, it is possible that API5 contributes to cancer metastasis through β-catenin- and ERK-mediated MMPs expression (Figure 4).

Another mechanism by which API5 contributes to tumor progression is through the induction of tumor immune escape. Indeed, in a very interesting study, the group led by Tae Woo Kim demonstrated that API5 plays key roles in both tumor progression and immunity [31]. Using different murine cancer models, Kim and colleagues showed that API5 could render tumor cells resistant to immune-mediated cytotoxicity, through the inhibition of tumor-specific T-cell-mediated apoptosis [31]. Mechanistically, API5 hinders T-cell-triggered apoptosis of cancerous cells by the upregulation of FGF-2 and subsequent activation of the FGFR1–PKCδ–ERK pathway, resulting in the ubiquitin-dependent degradation of the BH3-only protein BIM (Figure 3). Although these observations need to be confirmed using primary samples, they fit well with previous data indicating that cancer cells with high levels of AKT/ERK exhibit suppressed BIM expression [121,122], and they identify API5 as an immune-related prognostic biomarker. Therapeutic targeting of API5 could therefore represent a potential treatment option for cancer through tumor immune escape.

Finally, a substantial number of studies have shown that API5, which was initially identified for its antiapoptotic function, is critically involved in tumor survival and resistance to chemotherapeutic drugs. Morris and colleagues initially noted that the depletion of API5 was specifically lethal to tumor cells with deregulated E2F1 [39]. Shortly after, a crucial role for API5 in tumor cells’ sensitivity to anticancer drug was demonstrated by different groups. Indeed, the silencing of API5 in various cell lines sharply increased tumor cells’ sensitivity to chemotherapeutic drugs such as etoposide, camptothecin, or cisplatin, whereas API5-forced expression endowed cancer cells with enhanced resistance to these agents [26,65,123]. Although more research is needed to completely decipher the mechanisms at play, API5-induced resistance to anticancer drugs has been shown to stem from its activation of the FGFR1 signaling, which triggers the ERK-mediated degradation of the proapoptotic protein BIM, as well as the inhibition of caspase-2 and apoptotic DNA fragmentation [26,51,65,123] (Figure 3). Recently, API5 silencing has been linked to a sharp increase in cell death of caspase 9^−/−^ Jurkat cells treated with ABT-263, a potent and selective inhibitor of Bcl-2 and Bcl-xL [124]. Interestingly, ABT-263 is known to synergize with chemotherapies inducing DNA damage [125,126]. As the silencing of API5 promotes ABT-263-induced DNA damage [124], it is possible that API5 could function as a regulator of the DNA repair machinery, as its association with the chromatin remodeler ALC1 (amplified in liver cancer 1), which plays a key role in DNA repair, suggests [49]. In line with this hypothesis, API5 has been shown to be upregulated by UV irradiation of primary liver cells, and an increased expression of API5 protects primary liver cells from UV-induced apoptosis and to increase glioblastoma cells to radioresistance [127,128,129].

Combined, these data demonstrate a crucial role for API5 in cancer cell development and progression, providing a rationale for the therapeutic targeting of API5 for cancer treatment.

### 4.3. Targeting API5 as a Therapeutic Approach

Cancer is a consequence of multiple deregulated processes that endow tumor cells with certain traits which were described as “Hallmarks of Cancer” by Hanahan and Weinberg two decades ago [130]. Numerous new potential cancer targets have been identified over the last few years, and survival pathways, angiogenesis, DNA damage response (DDR), senescence pathways or the immune system, for instance, are important types of targets for the development of anticancer drugs. Clearly, given the above-described functions of API5, targeting this intriguing protein could be of great interest for cancer treatment. Among the different opportunities to indirectly or directly target proteins are their inhibition at the expression level, their inhibition through physical degradation or their inhibition at the protein/protein interaction level.

The downregulation of API5 expression has been achieved so far by means of RNA interference (RNAi), short hairpin RNAs (shRNAs), or microRNA (miRNA), and all these approaches have demonstrated interesting potentialities as they have resulted in cancer cells death, increased sensitivity to anticancer agents or immune-mediated cytotoxicity or inhibition of metastasis potential (see above). Therefore, RNA-based therapeutics approaches for API5 expression targeting could open novel possibilities for cancer treatment. However, critical challenges in applying these RNA therapies, related to pharmacodynamics and pharmacokinetics as well as immunogenicity issues, have hindered the clinical progress of RNA-based drugs [131]. Nonetheless, a substantial number of RNA-based therapeutics are currently under clinical investigation for various diseases, including cancers, and several RNA-based medications have been approved for clinical use [132]. Therefore, further research on RNA-based therapeutics for API5 targeting might lead to more RNA-based therapeutics for cancer treatment.

Direct API5 degradation is another therapeutic option. API5 stability has been demonstrated to be regulated via acetylation at lysine 251 (K251) by the histone acetyltransferase p300, which leads to an increase inAPI5 stability, whereas deacetylation by the histone deacetylase 1 HDAC1 reduces API5 levels [53]. Consequently, chemical inhibition of p300 resulted in decreased API5 levels, affecting its functions in cell cycle [53]. Interestingly, the expression of an acetylation-deficient mutant of API5 (K251A) did not protect tumor cells from apoptosis induced by serum deprivation [40]. Furthermore, tumor cells expressing API5 K251A in an API5 knockdown background could not survive while in culture [53]. Therefore, one can envision that the use of p300 inhibitors could constitute an interesting therapeutic option for the induction of the direct degradation of API5. As a matter of fact, the development of p300 inhibitors has attracted great attention in recent years due to its potential therapeutic value in the treatment of cancers [133,134]. Consequently, the steady-state API5 acetylation–methylation equilibrium, which functions as a molecular rheostat governing API5 stability and antiapoptotic properties, might be amenable to therapeutic exploitation as an anti-cancer strategy.

Finaly, the inhibition of API5 interactions with its partner proteins is another approach to target its biological functions. API5 interacts with several apoptosis-related proteins, and this complex-forming ability—probably favored by its elongated 3D structure [40]—appears to be essential for API5 to fulfill its antiapoptotic or metastatic functions [25,34,48,49,65]. Among its different domains, API5 contains several protein–protein interaction modules, such as HEAT and ARM repeat or the heptad leucine repeat region (Figure 1). Our group has shown that the heptad leucine repeat region of API5 mediates its interaction with several of its partners, such as Acinus and the kinase p21-activated kinase 1 (PAK1) [54,65]. Moreover, mutations of conserved residues (leucines 384 and 391 or arginine 382) in this domain abrogate API5 biological functions and prevent its interaction with its molecular partners [25,34,48,54,65,134]. This indicates that the heptad leucine repeat region of API5 could constitute a therapeutic target for anti-cancer drugs. We have constructed two API5-derived cell-permeable peptides, called RT53 and RT39, that comprise portions of the heptad leucine repeat region of API5 fused to a cell-penetrating sequence [54,65]. Both peptides acted as decoys and were able to prevent interaction between API5 and Acinus or PAK1 [54,65]. Moreover, the peptides demonstrated potent pro-apoptotic activity and synergy with anticancer drugs, as well as anti-migration potential, on multiple cancer cell lines as well as primary cutaneous T-cell lymphoma (Sézary syndrome) cells, thus phenocopying the consequences of API5 silencing [54,65,135,136,137]. The peptides also demonstrated in vivo efficacy as single agents in murine models of melanoma, breast cancer, acute promyelocytic leukemia, and Sézary syndrome, with very favorable half-lives in mice [27,54,65,135,136,137,138,139]. Structurally, RT53 and RT39 adopt a helical conformation, with an N-terminal stretch of arginine and lysine residues followed by a hydrophobic region, making them amphipathic and membrane active, similarly to other know membranolytic peptides [140]. Therefore, while sparing normal cells, the RT53 and RT39 peptides also possess oncolytic properties [54,135,136,137,139]. Mechanistically, RT39 retention in the membrane of Sézary cells is dependent on binding to PAK1 at the level of the plasma membrane, where PAK1 is strongly expressed [54,135]. Interestingly, oncolysis mediated by RT53 exhibited the hallmarks of immunogenic cell death, and vaccines consisting of APL or melanoma cells exposed in vitro to RT53 induced prophylactic and therapeutic protection in syngeneic murine models [136,137]. Therefore, RT53 and RT39 peptides’ anti-cancer action stems from both their ability to prevent API5 biological functions, through protein–protein interaction inhibition, and through their oncolytic properties.

Recently, the crystal structure of the API5–FGF-2 complex has been solved, allowing for the determination of the precise domains of the proteins involved in their interaction [47]. Based on this knowledge, Bong and colleagues have developed lentiviruses expressing a peptide composed of API5 residues 183–191, a domain involved in API5 interaction with FGF-2. Interestingly, the lentivirus-mediated expression of the API5-derived peptide in HeLa cells abrogated API5–FGF-2 interaction and reduced the nuclear export of bulk RNA, which is dependent on the API5–FGF-2 complex [47]. While it remains to be determined whether this novel API5-derived peptide exhibits anticancer effect or synergizes with anticancer drugs, these data support the testing of API5-derived peptides for cancer treatment.

## 5. Conclusions and Future Directions

Resistance to apoptosis is an undisputed factor in cancer development. Recent data indicate that API5, a multifunctional regulator of cell fate, clearly constitutes a protein of significant interest due to its crucial roles in cancer cells’ vital cellular processes, including, but far from being limited to, resistance to apoptosis. The transformed phenotype sustains uncontrolled proliferation, avoids cell death, and allows dissemination in secondary organs. API5 is a cornerstone of these tumoral phenotypes as it is involved in cancer cells’ survival, growth, metastasis, immune silencing, and resistance to chemotherapies, which represent excellent therapeutic targets. As such, the therapeutic inactivation of API5 functions for anti-cancer therapies is attractive, with recent work on API5 inhibitors identifying compounds to target its cellular functions. API5 acts as a scaffold protein, operating with multiple partners through protein–protein interactions (PPIs) that promote and support cancer cells’ pathological state. Among the challenging drug discovery tasks, one of the most complex and relevant tasks concerns drugs that interfere with PPIs. PPIs are essential to many biological processes, and a wealth of studies have shown that aberrant PPIs are associated with the progression of various disease states, including cancer, infectious diseases, and neurodegenerative diseases [141,142,143]. PPIs’ dynamic nature and the involvement of large protein surfaces are formidable obstacles that need to be overcome. However, recent results have revealed that the long-held dogma that protein–protein interactions are “undruggable” is being contradicted by a large body of work, and in recent years, several PPIs’ modulators have entered clinical studies, with a few drugs being placed on the market, indicating that such compounds have broad prospects in disease diagnosis and therapeutics [144,145]. The development of API5-derived peptides that are able to act as decoys to prevent API5 association with its protein partners’ functional interactions—hence shutting down API5 pro-tumoral functions—offers interesting prospects in the therapeutic modulation of this intriguing protein. Furthermore, these peptides have shown activity and efficacy in vivo in several xenograft models, serving as proof of concept of the therapeutic value of API5 inhibition. While its biological functions are far from being completely understood, numerous data clearly indicate that API5 has great potential as an intervention target for the novel treatment of refractory cancers, and its targeting can hence be regarded as a promising strategy in drug discovery.

## Figures and Tables

**Table 1:** Validated direct interactors of human API5.

Protein	Gene Name	Biological Impact	Reference
Fibroblast growth factor 2	*FGF2*	Modulation of mRNA nuclear export	[47,48]
Apoptotic chromatin condensation inducer in the nucleus	*ACIN1*	Regulation of apoptotic DNA fragmentation	[48]
Amplified in liver cancer 1	*ALC1*	Not determined	[49]
Nucleoprotein of influenza A virus	*NP*	Stimulation of E2F1- mediated apoptosis	[50]
Caspase-2	*CASP2*	Inhibition of caspase-2 activation	[51]
Estrogen receptor α	*ERα*	Gene expression regulation	[26]
Toll-like receptor 4	*TLR4*	Modulation of TLR4 signaling (agonist effect)	[52]
Leucine-rich pentatricopeptide repeat containing	*LRPPRC*	Modulation of mRNA nuclear export	[47]
U2AF65-associated protein 56	*UAP56*	Modulation of mRNA nuclear export	[47]
P300	*P300*	Regulation of API5 stability	[53]
Histone deacetylase 1	*HDAC1*	Regulation of API5 stability	[53]
p21-activated kinase 1	*PAK1*	Not determined	[54]
VP3 protein of Avibirnavirus	*VP3*	Regulation of API5 SUMOylation	[55]

## Data Availability

No new data were created.
